# Influence of students’ personality on their leisure behaviour choices and moderating effects on their academic efficacy: An exploratory study

**DOI:** 10.1371/journal.pone.0280462

**Published:** 2023-01-13

**Authors:** Susen Köslich-Strumann, Christoph Strumann, Edgar Voltmer

**Affiliations:** 1 Institute of Social Medicine and Epidemiology, University of Lübeck, Lübeck, Germany; 2 Institute of Family Medicine, University Hospital Schleswig-Holstein, Lübeck, Germany; COMSATS University Islamabad - Wah Campus, PAKISTAN

## Abstract

Studying can be very stressful leading to a decreased academic efficacy. In this exploratory longitudinal study, we analysed a wide range of students’ leisure activities and their effects on students’ academic efficacy. Further, we identified the personality types of students who choose specific leisure activities as a strategy to stress reduction and determined how the use of leisure behaviours affects academic performance among students with different personality types. Students were asked about their personality (Neo-FFI), leisure time behaviour (self-generated items), and academic efficacy (MBI-SS) at three measurement points. Multivariate regression analyses were applied to estimate the moderation effects. In total, 331 students were included in the study. Social activities were found to have a direct effect on academic efficacy. The students’ personality moderated the effects of leisure behaviour on efficacy, suggesting a negative effect on academic efficacy for some personality traits. Since our results suggest that the effectiveness of stress management through the use of leisure behaviour depends on the students’ personality, universities offering stress management services should pay attention to precise targeting to attract the specific students who might benefit the most from the offered services.

## Introduction

Studying is perceived as a stressful task by many students [1,2]. Students’ stress factors include exams, academic performance, success, graduation planning, and time pressure [3–5]. The introduction of Bachelor’s (BA) and Master’s (MA) courses as part of the Bologna reform and the associated modularization of courses have further increased the burden on students in Europe [6] in the subjects concerned [5,7]. In addition, regarding their stage of life, students have to cope with new challenges and uncertainties, as for example the process of identity formation and organizational challenges associated with living alone for the first time [4,8].

Chronic stress exposure can lead to psychological problems, such as depression and anxiety symptoms, which can increase the risk of suicide [9]. In many studies, students have shown increased scores for burnout [1,10]. The high demands of studying leads students to feel emotionally exhausted and to adopt a cynical, distanced attitude toward the content of their studies. A feeling of being incompetent can further reduce the motivation to study leading to a performance deterioration [11] with increased perceived stress [12]. This vicious cycle can result in burnout and prompt students to abandon their studies [13].

Academic success and life satisfaction are positively associated with health-promoting strategies for coping with stress [14]. To cope with the stress of studying and thus to maintain or increase their performance during their studies, students use different stress management strategies, including the individual organization of leisure time behaviour according to their preferences [15]. As proposed by Bergmann et al. [8], leisure activities are considered as an important resource to facilitate the recovery from academic stress. However, research lacks a comprehensive empirical evaluation of the effectiveness of students’ individual leisure time behaviour on their ability to deal with stress. Studies have usually focused on separate activities, such as gaming, drinking, or sports [16–18].

Previous studies have found a relationship between personality and stress perception [19] as well as the selection of coping strategies [15,20]. However, they have not considered students’ personalities in relation to stress management and academic efficacy. For instance, since extroverted persons are more willing to participate in sports during their leisure time [21], the positive impact on their stress reduction level might be higher for these persons than for introverted people. Therefore, we expect that leisure behaviours that fit with individuals’ personality have a stronger impact on their ability to deal with stress. The consideration of the students’ personality could be used for designing stress management programmes to be particularly effective for students, enabling their experience of studying to be as effective and stress free as possible.

Against this background, this exploratory study analysed a wide range of students’ leisure activities and their effects on students’ academic efficacy. We also identified the personality types of students who choose specific leisure activities as a strategy to stress reduction and determined how the use of leisure behaviours affects performance among students with different personality types.

## Methods

### Participants

Data were drawn from an ongoing longitudinal observational study at the University of Lübeck (UzL) [22]. The Lübeck University Students Trial (LUST) has been surveying students at the University of Lübeck about their health every year since 2011 [23–25]. All participants provided written informed consent. The LUST study addressed all students at the University of Lübeck; i.e., the inclusion criterion was enrollment at the UzL; there were no exclusion criteria. A detailed description of the LUST study can be found in the report by Kötter et al. [23]. The UzL is a public, life science-oriented university with approximately 5000 enrolled students. Medical students form the largest study group, with about 1600 students [22].

A total of 331 students answered the questions on their leisure time behaviour to cope with stress and the items on the personality traits and the MBI-SS and could thus be included in the study. Table 1 shows the descriptive sample characteristics. The median age was 22 years, and the majority of the students were female (74%). More than half of the students studied medicine (55.3%).

**Table 1:** Sample characteristics.

Variable	N (%)
*Sociodemographic*	
female	245 (74.0)
male	85 (25.7)
diverse	1 (0.3)
age (years), median (IQR)	22 (2)
*Subject*	
medicine	183 (55.3)
STEM	114 (34.4)
health science	34 (10.3)
*Freshmen in*	
2011	75 (22.7)
2012	91 (27.5)
2016	165 (49.9)
*Intrinsic motivation*	
studying is most important *((1)*: *not true at all*,.* *.* *., *(5)*: *is totally true)* mean±std	3.12±0.86
*Maslach Burnout Inventory*	
efficacy	4.89±1.00

IQR: Interquartile; std: Standard deviation.

### Instruments and measurements

#### Personality

The personality traits of the students were measured using the five-factor model of Costa and McCrae [21,26]. The Big Five model describes an individual’s personality with five factors, which in turn are made up of different facets. The factors with their facets are neuroticism (e.g., fearfulness, impulsiveness, and vulnerability), extraversion (e.g., activity and assertiveness), openness to experience (e.g., fantasy, feelings, and ideas), conscientiousness (e.g., orderliness, sense of duty, and prudence), and agreeableness (e.g., trust, altruism, and kindness) [27]. The short version of the Neo-FFI instrument assessing the Big-Five personality dimensions [28] was surveyed in the baseline year at t0. The score for each dimension ranges from 1 to 5. Since people’s personality is expected not to change in the short run, we used the measured scores at t0 for the analyses of the other measurement times. The reliability of the NEO-FFI were measured in our sample as Neuroticism: 0.83; Extraversion: 0.76; Openness: 0.79; Conscientiousness: 0.75 and Agreeableness: 0.70. The internal consistency of the total scale was 0.80. These numbers were similar to related studies [28].

#### Stress management through choice of leisure behaviour

We operationalized the stress management behaviour by asking students about their preferred extracurricular activities for the purpose of stress reduction in the second year of studying (t2). Besides 18 items covering specific activities, for example, participating in sports, meeting friends, and smoking, students were able to respond in free-text format. These answers were categorized into media, social, relaxation, exercise, creativity, harmful, and religious activities.

The category formation was based on the following considerations and findings.

*Media*. Middendorf et al. [29] also formed categories from the options for dealing with stress caused by the pressure to perform during studies. They created, among others, the category media, computer games, and television. We expanded this category and included watching TV, surfing the internet, reading, listening to music, and gaming in our media category.

*Social*. Middendorff et al. [29] found that social contacts, for example friends, are important for coping with the demands of studying. They formed the social network category. Hess and Copeland [30] also identified a positive correlation between social activities and coping with stress among students. Based on these findings, we formed our social category and included meeting friends, as well as activities that take place primarily in social contexts, as talking on the phone, and partying.

*Relaxation*. This category includes sleep and relaxation techniques. Both have similar relaxing effects, such as muscle relaxation, regular breathing, and increased efficacy on everyday life [31,32]. In addition, relaxation techniques (as e.g., progressive muscle relaxation or autogenic training) are used successfully to treat sleep disorders [32].

*Exercise*. We also created the category exercise that includes sports and going for a walk. Middendorf et. al [29] found that sports are a single dimension to cope with stress for students. In addition to sports, even moderate exercise, such as going for a walk, has a positive effect on mental well-being [33].

*Creativity*. The creativity category includes making music, singing, and engaging in handicrafts. The meta-analysis by Martin et al. [34] showed that 81.1% of the included studies reported a significant reduction in stress among participants when the interventions included singing and/or artistic activities.

*Harmful*. Furthermore, we formed the category ‘harmful’, which consists of eating, drinking alcohol, smoking, and taking drugs/medication. The category summarizes all those behaviours that can damage health. Smoking as well as alcohol and drug consumption/medication can damage health [35]. In the context of our analyses, we classified eating as a harmful behaviour, because the item asked for the intentional use of eating for stress management, and various studies have shown the negative effect of stress-induced eating. For instance, Macht [36] reported that stress increases eating habits if this has the function of reducing negative emotions. The study by Oliver et.al [37] showed that stressed emotional eaters ate more sweet, high-fat foods and higher-energy meals than unstressed and non-emotional eaters.

*Religious*. We also formed the category ‘religious’. Students who expressed spirituality through religious beliefs had higher immunity to stressful situations than students who identified as spiritual but not religious [38].

#### Academic efficacy

To evaluate the academic efficacy of the students, we used the Maslach Burnout Inventory (MBI) adapted for use among students (MBI-SS) [11]. The instrument is composed of 15 items assessing the three dimensions of burnout, specifically exhaustion (five items), cynicism (four items), and efficacy (six items). The 15 items are mapped onto a 7-point Likert scale (1 = never to 7 = always). High scores on the exhaustion and cynicism scales and low scores on the efficacy scale indicate a risk of burnout [39,40]. We concentrated the analysis on efficacy, since people who show high scores on this dimension tend to be high achievers. This is of particular relevance to students’ successful completion of their study [41,42]. For this study, data from the MBI-SS in the third year of study (t3) were used. The internal consistencies of the MBI-SS according to Cronbach’s alpha were in this sample α = 0.83 and, thus, similar to other studies [40].

#### Procedure

While the students’ first survey (baseline, t0) was conducted with a paper-pencil procedure in class during the pre-course week, the follow-up surveys took place online in June during the respective summer semesters. As an incentive to participate, the students received a 5€ voucher for each completed questionnaire.

This study was conducted in accordance with the guidelines provided by the Declaration of Helsinki. The study protocol was approved by the Ethical Committee of the University of Lübeck. The study protocol and a comprehensive self-report questionnaire were submitted to the UzL ethics committee for review and discussed by the committee members at a meeting. In the study protocol, the methodological procedure, included variables, outcomes, as well as study consent and data protection procedures were described and checked. In the course of the study, in the case of significant adjustments to the LUST study with regard to variables, requests for updates were submitted to the ethics committee with a supplementary study protocol in each case. Based on this information, the ethics committee then gave a positive vote (file reference: 11–010).

#### Motivation

We measured the motivation of the students with the following item from the instrument Work-Related Behaviour and Experience Pattern (AVEM) [43]: ‘For me, studying is the most important part of life’, ranging from 1 (not correct at all) to 5 (applies completely).

### Data analysis

For this study, three cohorts of medical, health science, and science, technology, engineering, and mathematics (STEM) students (freshmen in 2011, 2012, and 2016) were analysed in their first 3 years of study. For these cohorts, the survey applied the questionnaires that are relevant to our study in the longitudinal design.

In the first step, we investigated whether there are differences between the students’ personality traits and the leisure behaviour that they apply to manage stress. For this purpose, we computed the mean of the five personality dimensions for groups applying a specific leisure behaviour or not. The differences were tested for significance by means of the t-test.

In the second step, we tested whether the students’ personality moderates the effects of the distinct leisure behaviours on the efficacy in the next year. We applied regression analysis with efficacy serving as the dependent variable. Six dummy variables covering the categorized leisure behaviour (active, social, media consumption, relaxation, harmful activities, and religious engagement) in the second year (t2) are of key interest in explaining the efficacy in the third year (t3). The time lag is used to reduce the risk of reverse causality. To analyse the moderation of these effects by the personality traits, we introduced corresponding interaction effects.

Further, we controlled for the sex, age, and study cohort and whether the students were enrolled in medical study. This is important here because medical students make up a disproportionately large part of the sample [22]. The motivation of the students was also considered as a control variable to reduce the risk of biased results due to the omission of potential confounder variables.

Statistical analyses were performed with STATA 15 (StataCorp LLC, College Station, TX, USA).

## Results

The mean evaluation of the importance of studying (motivation) was 3.12, and the average efficacy of the MBI efficacy score was 4.89. This value indicates a low burnout value for the students. We interpreted the values based on Celik and Oral [44].

### Leisure behaviour and personality

In Table 2, we display the distribution of students with different types of leisure behaviour and the average scores of the five personality dimensions. In addition, for each behaviour, the differences between these scores for students who are pursuing or not pursuing the respective leisure behaviour are shown. Students who were using exercise for stress reduction had higher scores for extraversion. However, within the individual strategies, there are differences. While students engaging in sports had higher scores for extraversion and conscientiousness, going for a walk was associated with higher openness scores. Similarly, higher openness scores were observed for students pursuing creative activities. These students were also more agreeable and neurotic. Meeting friends was associated with extraversion and agreeableness, while watching TV was an activity carried out by less extraverted and less open persons. Students that pursue gaming had significantly lower scores for extraversion, agreeableness, and conscientiousness.

**Table 2:** Leisure behavior and personality.

Leisure behavior to manage stress			Conscientiousness		Extraversion		Openness		Agreeableness		Neuroticism	
	n = 331		mean:	4.07	mean:	3.51	mean:	3.69	mean:	4.02	mean:	2.54
	yes (%)	no (%)	Δ(*y*/*n*)	p-val	Δ(*y*/*n*)	p-val	Δ(*y*/*n*)	p-val	Δ(*y*/*n*)	p-val	Δ(*y*/*n*)	p-val
**exercise**	84.9	15.1	0.10	0.203	0.25	**0.009**	0.16	0.142	0.12	0.153	-0.18	0.120
sports	71.0	29.0	0.18	**0.003**	0.22	**0.002**	-0.01	0.913	0.05	0.479	-0.16	0.074
going for a walk	48.6	51.4	0.05	0.376	0.05	0.441	0.29	**0.000**	0.09	0.148	0.03	0.750
**creative**	33.5	66.5	0.04	0.518	0.02	0.820	0.36	**0.000**	0.19	**0.004**	0.19	**0.028**
making music, singing	28.4	71.6	0.00	0.950	0.03	0.660	0.33	**0.000**	0.14	**0.042**	0.13	0.149
doing handicrafts	9.7	90.3	0.08	0.424	0.03	0.789	0.41	**0.003**	0.19	0.067	0.35	**0.014**
**social**	80.1	19.9	0.00	0.955	0.15	0.072	0.05	0.604	0.11	0.149	-0.04	0.713
meeting friends	69.5	30.5	0.00	0.959	0.15	**0.038**	0.03	0.716	0.15	**0.022**	-0.13	0.162
partying	10.0	90.0	-0.05	0.602	0.07	0.530	-0.12	0.360	-0.11	0.296	0.27	0.053
talking on the phone	39.0	61.0	0.02	0.662	0.09	0.192	0.13	0.107	0.03	0.681	0.04	0.601
**media**	92.1	7.9	-0.20	0.051	-0.17	0.171	-0.07	0.652	-0.03	0.811	0.08	0.611
going to the cinema	12.4	87.6	0.00	0.997	-0.07	0.464	0.05	0.678	0.02	0.799	-0.05	0.677
listening to music	68.3	31.7	-0.02	0.783	-0.08	0.267	0.10	0.243	0.16	**0.013**	-0.02	0.863
watching TV	52.3	47.7	0.03	0.617	-0.16	**0.016**	-0.25	**0.001**	-0.09	0.139	0.06	0.470
surfing the Internet	41.7	58.3	-0.16	**0.003**	0.01	0.855	0.01	0.916	-0.13	**0.030**	-0.07	0.403
reading	37.8	62.2	0.01	0.823	-0.08	0.243	0.09	0.259	0.06	0.323	0.00	0.962
gaming	14.8	85.2	-0.19	**0.015**	-0.31	**0.001**	0.03	0.781	-0.29	**0.001**	-0.19	0.104
**harmful**	55.6	44.4	0.01	0.791	0.04	0.601	-0.02	0.795	-0.04	0.550	0.02	0.853
eating	48.6	51.4	0.02	0.776	0.04	0.596	0.06	0.481	-0.03	0.569	0.06	0.497
drinking alcohol	13.9	86.1	-0.06	0.458	0.02	0.851	-0.13	0.252	-0.15	0.099	-0.04	0.752
smoking	3.6	96.4	0.00	0.996	-0.05	0.778	-0.03	0.880	-0.06	0.695	-0.25	0.267
taking drugs/medication	0.9	99.1	0.31	0.295	-0.12	0.736	0.10	0.807	0.04	0.911	0.12	0.781
**relaxation**	13.6	86.4	0.13	0.102	0.00	0.998	0.25	**0.031**	0.10	0.276	-0.03	0.829
using relaxation techniques	11.8	88.2	0.11	0.198	0.01	0.951	0.26	**0.034**	0.10	0.275	-0.01	0.955
sleep	1.8	98.2	0.23	0.279	-0.04	0.888	0.13	0.658	0.04	0.874	-0.13	0.677
**religious**	12.1	87.9	0.10	0.247	0.07	0.473	-0.08	0.534	0.11	0.220	0.09	0.470

Δ(*y*/*n*): Differences between personality scores for students pursuing or not pursuing the respective leisure behavior.

About 14% of the students used relaxation techniques and were more open than their fellow students. Religiousness was not associated with personality.

### Moderation analysis

To analyse the moderation of the effect of leisure behaviour on efficacy by the five personality traits, we estimated seven multivariate regression models. In the first model, no moderation effects were specified. The other five models included the moderation effects of each personality trait separately for all the leisure behaviour categories. The full model included all the moderation effects jointly. The estimation results are shown in Table 3 for the significant interaction effects (see S1 Table for the full list of interaction variables).

**Table 3:** Regression analysis.

Variable	Baseline	Conscient iousness	Extra version	Open ness	Agreea bleness	Neuro ticism	Full
***controls***							
intercept	2.27***	2.08**	1.77**	2.42***	2.28***	2.17**	1.41
female	-0.08	-0.09	-0.1	-0.08	-0.06	-0.05	-0.07
age	-0.01	-0.01	-0.01	-0.01	-0.01	-0.01	-0.01
medicine	0.33***	0.34***	0.39***	0.31***	0.31***	0.35***	0.41***
starting year: 2012	0.02	0.01	0.05	-0.02	0.03	0.01	0.02
starting year: 2013	-0.03	-0.02	0.05	-0.07	-0.05	-0.03	0.04
*(reference*: *2016)*							
studying is most important	0.27***	0.27***	0.25***	0.27***	0.26***	0.27***	0.25***
***leisure behaviour (previous year)***							
exercise	-0.03	-0.02	0.10	-0.07	-0.01	-0.06	0.03
creative	0.00	0.00	0.02	0.00	0.02	0.01	0.07
social	0.28**	0.29**	0.31**	0.31**	0.30**	0.30**	0.38***
media	-0.3	-0.26	-0.13	-0.34*	-0.3	-0.26	0.07
harmful	-0.13	-0.12	-0.15	-0.12	-0.12	-0.12	-0.11
religious	0.11	0.09	0.18	0.03	0.12	0.1	0.00
relax	-0.12	-0.15	-0.17	-0.11	-0.1	-0.11	-0.08
***personality***							
conscientiousness (C)	0.33***	0.33***	0.34***	0.28***	0.33***	0.32***	0.29***
extraversion (E)	0.17*	0.16*	0.20**	0.19**	0.16*	0.16*	0.24***
openness (O)	0.05	0.07	0.06	0.03	0.06	0.07	0.07
agreeableness (A)	0.17*	0.17*	0.16*	0.21**	0.17*	0.16*	0.21**
neuroticism (N)	-0.32***	-0.32***	-0.33***	-0.34***	-0.31***	-0.33***	-0.35***
***interaction terms***							
E & exercise			0.53**				0.56**
E & creative			0.34*				0.55***
E & media			-0.91**				-1.28***
E & relax			0.53**				0.80***
O & media				-0.34			-0.56*
O & harmful				0.20			0.33**
O & religious				-0.56**			-0.64**
O & relax				-0.29			-0.50**
N & creative						0.12	0.26*
N & relax						0.21	0.43**
LOGLIKE	-415.2	-414.1	-404.2	-409.4	-411.6	-412.5	-381.7
AIC	868.3	880.2	860.4	870.8	875.1	877.0	871.4
BIC	940.6	979.0	959.3	969.7	974.0	975.9	1076.7

Notes: Efficacy Serves as the dependent variable; based on 331 observations; significance levels

* 10%

** 5%

*** 1%; LOGLIKE: Log likelihood value; AIC: Akaike Information Criterion; BIC: Bayesian Information Criterion.

All the estimated coefficients of the direct effects are rather stable over the different model specifications. Regarding the baseline model, medical students and students with high motivation had a higher level of efficacy. Differences between gender, age, and starting year cannot be detected.

While neuroticism is negatively correlated with efficacy, conscientiousness has a positive association. Extraversion and agreeableness are only significant at the 10% level. Pursuing social activities for stress reduction in the previous year was associated with increased efficacy. For the other behaviours, no direct impact could be observed.

However, the effects of some of these behaviours on efficacy were moderated by personality. The effects of exercising in leisure time and the application of relaxation techniques on efficacy were both significantly higher for extraverted students. In contrast, these students faced significantly lower effects of media consumption on efficacy. Relaxation and religiousness both had negative impacts on efficacy for open students.

## Discussion

Studying can be very stressful. As a result, performance can decrease and even burnout can be a threat. To prevent high stress levels and a decline in academic efficacy, students use different stress management strategies; one of them is to engage activities in their leisure time. In this study, we examined the influence of personality on leisure behaviour choices. Further, we investigated the moderation of the effects of leisure behaviour on efficacy by students’ personality.

### Extraversion

Our results suggest that students who participate in sports or meet friends for stress reduction are more extraverted, while watching TV and gaming seem to be applied by students with low extraversion scores. Extraversion is described by the facets of activity, sociability, warmth, and hunger for experience, among others [45]. People with high extraversion values in general have more friends and take part in more sports than people with low extraversion levels [21]. Further, extroverts tend to handle their emotions by seeking social support [46]. This is in line with our results, since sports and meeting friends are active, stimulating, and energizing and acts of socializing (with the exception of performing sports alone). Watching TV and gaming might be perceived as boring and not stimulating enough for extraverts, who seek company and like to be active. Our finding that extraverted students have higher efficacy has also been observed in the literature [47,48]. Further, for these students, participating in sports and meeting friends seem to be effective in coping with stress. Students who engage in sports regularly feel better socially supported and use sports as a positive stress management strategy [17]. Another stress management strategy for extraverted students might be the application of relaxation techniques, which have generally been found to have positive effects on stress reduction [49]. Because both the use of sports [50] and the use of relaxation techniques have a positive effect on coping with stress [31], it is conceivable that the combination of the two has a positive effect enabling students to increase their performance and cope with stress. The application of this effective combination could be one reason for extraversion being positively associated with performance and stress resistance. On the contrary, if extraverted students use media or apply harmful strategies, the respective effects on efficacy are smaller (i.e., more harmful) than those for students with low extraversion scores. The use of media or harmful behaviour leads to increased distraction from learning and promotes procrastination [51]. Watching TV and surfing the Internet are often time consuming, and one can be seduced by always new incentives to continue, which can distract one and cost further time that one could use for learning. Harmful behaviour like smoking and drinking can have a negative effect on the physical condition, reducing the academic performance [52].

### Openness

Our results further show that students scoring high on openness prefer to engage in a variety of leisure activities. This was also observed by Costa and McCrae [21]. Open people are generally interested in new experiences [45]. Students who watch TV to reduce stress have lower openness scores. For open students, this activity could be perceived as not having enough variety. Although we did not find a direct effect of openness on efficacy, others have observed a negative correlation between openness and stress, suggesting higher stress resilience of open people [53]. The application of relaxation techniques is found to have a negative effect on stress reduction for open people. Since open people are more easily distracted and less able to concentrate [54], the effectiveness of the relaxation technique might be reduced. On the other hand, it could also be argued that open people are inquisitive and thus enjoy learning.

### Neuroticism

People with high neuroticism scores are considered to be anxious and emotionally unstable and to have little resistance to stress [21,45]. This is in line with our finding that neurotic students have lower efficacy levels. This correlation has also been described in the literature [55–57]. People with high levels of neuroticism tend to behave impulsively, without thinking twice about the potential negative impacts on their academic performance [46]. Further, students who engage in handicrafts for stress reduction have higher neuroticism scores. Handicrafts might provide security and a sense of control over something that is manageable, with direct enjoyment of the results [58]. Moreover, handicrafts are a conflict-free field and there is no need for social interaction that might upset neurotic people [59]. Handicrafts require concentration solely on one thing and can thus occupy one’s full attention, preventing negative thoughts and worries, which are typical for emotionally unstable people [21,45,60]. In a systematic review, Dunphy et al. [61] listed several studies that found a positive effect of creative arts interventions on depression.

### Conscientiousness

Conscientious people were found to engage more often in sports in our study, which is consistent with the findings of others. Bogg and Roberts [62] described a negative correlation between conscientiousness and unhealthy behaviours (e.g., an unhealthy diet and alcohol and drug use). The increased efficacy of conscientiousness in our study has also been approved in the literature [55]. This result is consistent with the fact that striving for performance is one of the facets used to describe conscientiousness, and conscientious people are considered to be dutiful, to be disciplined, and to plan ahead [45]. The insignificance of the moderation effects of conscientiousness might be explained by the high performance level of conscientious persons. They perform well anyway due to their personality traits, and consequently their choice of leisure behaviour might not influence their already high performance, suggesting that they do not explicitly need stress management to increase their performance.

### Agreeableness

People with high levels of agreeableness are characterized by facets of trust, responsiveness, and helpfulness [45]. This might explain the higher scores for students who participate in music and singing, meet friends, and listen to music. In contrast, surfing the Internet and gaming are carried out by persons with less agreeableness. Efficacy was found to be higher for students with high levels of agreeableness.

### Implications for research and practice

Universities have implemented various approaches to provide supportive and ‘health-promoting’ environments [63]. Programmes that are offered by universities, for example sports programmes to increase resilience or attention training, have already been examined and evaluated [64–67]. Since our results suggest that the effectiveness of stress management strategies depend on the students’ personality, universities should offer leisure time activities as individually as possible. They should also pay attention to precise targeting to attract the specific students who might benefit the most from the offered methods [68]. Using a ‘wrong’ leisure activity as a stress management method could lead to more stress and reduce students’ efficacy. One possible starting point for designing individual stress management strategies could be to consider the students’ personalities. These could be surveyed in advance, for example by asking for preferences and then offering appropriate courses to students in a targeted manner. Future studies should investigate whether targeted courses have greater effectiveness among students.

### Strengths and limitations

The present study has strengths and weaknesses. It is one of the few long-term designs on the topic of leisure behaviour, personality, and performance among students. Another strength is that it considers a wide range of leisure activities and thus can provide an overview of which leisure activities students practise depending on their personality. Since the University of Lübeck has mainly STEM and medical students, the representativeness of the sample is limited. Moreover, women and medical students are overrepresented in comparison with the general student body in Germany. Furthermore, there might be a selection bias since the participation in the survey was voluntary. Therefore, the possibility cannot be excluded that only students who were intrinsically motivated participated in the survey. However, incentives in the form of vouchers might also have created an extrinsic motivation to participate.

The exploratory character of this analysis and the absence of a coherent theoretical construct prevented us to draw causal conclusions from the data. However, our findings that the students’ personality moderates the effects of leisure behaviour on academic performance might be helpful to develop a coherent theoretical framework that can be used in future studies. Moreover, the personality factors were not related to the level of regulation of the students. The regulatory factors of the context should be used in future research. Further, based on a coherent theoretical framework, future studies might investigate a mediation model to identify the causal effect of the students’ personality on academic performance via applying different coping strategies.

The estimation of the moderation effect of the students’ personality was based on separated personality traits. A differential personality profile was not delimited. As a consequence, the estimated effects may be subject to overlap.

## Conclusion

In this exploratory study, we contribute to the literature by identifying personality types of students who choose specific leisure activities as a strategy to stress reduction. Further, we determined how the use of leisure behaviours affects performance among students depending on their personality type.

Our findings suggest that for extraverted students, doing sports, meeting friends and applying relaxation techniques seem to be effective in coping with stress. The choice of leisure activity can also have negative effects on academic efficacy for some personality traits. For open people, the application of relaxation techniques is found to have a negative effect on stress reduction. Our results suggest that universities should offer their health promotion/stress management programmes in a more personalized way to address students selectively. In this way, stress management/health promotion services could be more effective in maintaining or even improving students’ academic performance. Universities could offer interventions targeting specific personality types. For instance, group sport offers that particularly appeal to extroverts. Offering in small groups (e.g., handicrafts) might be suitable for students with a high neuroticism value, due to less stress through social interaction. Students could complete a personality questionnaire and interests screening before they participate in an intervention tailored to their personality traits and interests.

Another possibility for an intervention in an academic context could be an elective or an extracurricular offer that includes psychoeducation, in which the different needs of the individual personalities could be explained, which the students could then take into account when choosing their leisure behaviour to relieve stress. In this way, choosing the appropriate leisure behaviour as stress management strategy could contribute to better and more effective stress relief.

## Supporting information

S1 Table Regression analysis (with full list of interaction variables). (DOCX)

## Abbreviations

NEO-FFI: NEO-Five-Factor Inventory
MBI-SS: Maslach Burnout Inventory
LUST: Lübeck University Students Trial
UzL: University of Lübeck
STEM: science, technology, engineering, and mathematics
