# Supplementary material for: Influence of students’ personality on their leisure behaviour choices and moderating effects on their academic efficacy: An exploratory study
Source: PLoS One. 2023 Jan 13;18(1):e0280462. doi: 10.1371/journal.pone.0280462 (PMC9838833; doi:10.1371/journal.pone.0280462)
Supplement: S1 Table — (DOCX) [file pone.0280462.s001.docx]

**S1 Table: Regression analysis (with full list of interaction variables)**

| **Variable** | **Baseline** | **Conscient**  **iousness** | **Extra**  **version** | **Open**  **ness** | **Agreea**  **bleness** | **Neuro**  **ticism** | **Full** |
| --- | --- | --- | --- | --- | --- | --- | --- |
| ***controls*** |  |  |  |  |  |  |  |
| intercept | 2.27*** | 2.08** | 1.77** | 2.42*** | 2.28*** | 2.17** | 1.41 |
| female | -0.08 | -0.09 | -0.1 | -0.08 | -0.06 | -0.05 | -0.07 |
| age | -0.01 | -0.01 | -0.01 | -0.01 | -0.01 | -0.01 | -0.01 |
| medicine | 0.33*** | 0.34*** | 0.39*** | 0.31*** | 0.31*** | 0.35*** | 0.41*** |
| starting year: 2012 | 0.02 | 0.01 | 0.05 | -0.02 | 0.03 | 0.01 | 0.02 |
| starting year: 2013 | -0.03 | -0.02 | 0.05 | -0.07 | -0.05 | -0.03 | 0.04 |
| *(reference: starting year: 2016)* |  |  |  |  |  |  |  |
| studying is most important | 0.27*** | 0.27*** | 0.25*** | 0.27*** | 0.26*** | 0.27*** | 0.25*** |
| ***leisure behaviour (previous year)*** |  |  |  |  |  |  |  |
| exercise | -0.03 | -0.02 | 0.1 | -0.07 | -0.01 | -0.06 | 0.03 |
| creative | 0 | 0 | 0.02 | 0 | 0.02 | 0.01 | 0.07 |
| social | 0.28** | 0.29** | 0.31** | 0.31** | 0.30** | 0.30** | 0.38*** |
| media | -0.3 | -0.26 | -0.13 | -0.34* | -0.3 | -0.26 | 0.07 |
| harmful | -0.13 | -0.12 | -0.15 | -0.12 | -0.12 | -0.12 | -0.11 |
| religious | 0.11 | 0.09 | 0.18 | 0.03 | 0.12 | 0.1 | 0 |
| relax | -0.12 | -0.15 | -0.17 | -0.11 | -0.1 | -0.11 | -0.08 |
| ***personality*** |  |  |  |  |  |  |  |
| neuroticism (N) | -0.32*** | -0.32*** | -0.33*** | -0.34*** | -0.31*** | -0.33*** | -0.35*** |
| extraversion (E) | 0.17* | 0.16* | 0.20** | 0.19** | 0.16* | 0.16* | 0.24*** |
| openness (O) | 0.05 | 0.07 | 0.06 | 0.03 | 0.06 | 0.07 | 0.07 |
| agreeableness (A) | 0.17* | 0.17* | 0.16* | 0.21** | 0.17* | 0.16* | 0.21** |
| conscientiousness (C) | 0.33*** | 0.33*** | 0.34*** | 0.28*** | 0.33*** | 0.32*** | 0.29*** |
| ***interaction terms*** |  |  |  |  |  |  |  |
| C & exercise |  | 0.19 |  |  |  |  | -0.04 |
| C & creative |  | 0.04 |  |  |  |  | -0.10 |
| C & social |  | -0.27 |  |  |  |  | -0.42 |
| C & media |  | -0.19 |  |  |  |  | -0.46 |
| C & harmful |  | 0.06 |  |  |  |  | 0.09 |
| C & religious |  | 0.16 |  |  |  |  | 0.17 |
| C & relax |  | 0.10 |  |  |  |  | -0.29 |
| E & exercise |  |  | 0.53** |  |  |  | 0.56** |
| E & creative |  |  | 0.34* |  |  |  | 0.55*** |
| E & social |  |  | 0.09 |  |  |  | 0.26 |
| E & media |  |  | -0.91** |  |  |  | -1.28*** |
| E & harmful |  |  | -0.20 |  |  |  | -0.28 |
| E & religious |  |  | -0.05 |  |  |  | 0.18 |
| E & relax |  |  | 0.53** |  |  |  | 0.80*** |
| O & exercise |  |  |  | -0.06 |  |  | -0.17 |
| O & creative |  |  |  | 0.01 |  |  | 0.03 |
| O & social |  |  |  | 0.09 |  |  | -0.13 |
| O & media |  |  |  | -0.34 |  |  | -0.56* |
| O & harmful |  |  |  | 0.20 |  |  | 0.33** |
| O & religious |  |  |  | -0.56** |  |  | -0.64** |
| O & relax |  |  |  | -0.29 |  |  | -0.50** |
| A & exercise |  |  |  |  | 0.33 |  | 0.20 |
| A & creative |  |  |  |  | -0.23 |  | -0.34 |
| A & social |  |  |  |  | 0.03 |  | 0.20 |
| A & media |  |  |  |  | -0.48 |  | -0.57 |
| A & harmful |  |  |  |  | -0.16 |  | -0.10 |
| A & religious |  |  |  |  | -0.09 |  | -0.07 |
| A & relax |  |  |  |  | 0.20 |  | 0.31 |
| N & exercise |  |  |  |  |  | 0.09 | 0.17 |
| N & creative |  |  |  |  |  | 0.12 | 0.26* |
| N & social |  |  |  |  |  | 0.06 | 0.16 |
| N & media |  |  |  |  |  | 0.18 | 0.07 |
| N & harmful |  |  |  |  |  | 0.18 | 0.06 |
| N & religious |  |  |  |  |  | -0.11 | -0.18 |
| N & relax |  |  |  |  |  | 0.21 | 0.43** |
| observations | 331 | 331 | 331 | 331 | 331 | 331 | 331 |
| LOGLIKE | -415.2 | -414.1 | -404.2 | -409.4 | -411.6 | -412.5 | -381.7 |
| AIC | 868.3 | 880.2 | 860.4 | 870.8 | 875.1 | 877.0 | 871.4 |
| BIC | 940.6 | 979.0 | 959.3 | 969.7 | 974.0 | 975.9 | 1076.7 |

Notes: Efficacy serves as the dependent variable; significance levels: * 10%, ** 5%, *** 1%; LOGLIKE: log likelihood value; AIC: Akaike Information Criterion; BIC: Bayesian Information Criterion.
